# Effects of *C*-Ring Structural Differences on the Inhibition of *N^ε^*-(Carboxyethyl)lysine in the Methylglyoxal-Lysine System by Flavonoids

**DOI:** 10.3390/ijms26125914

**Published:** 2025-06-19

**Authors:** Yating Ling, Linlin Zhang, Bangzhu Peng, Zhuo Zhang

**Affiliations:** 1 College of Food Science & Technology, Huazhong Agricultural University, Wuhan 430070, China; lilly@webmail.hzau.edu.cn (Y.L.); zhanglinlin@webmail.hzau.edu.cn (L.Z.); bzpeng@mail.hzau.edu.cn (B.P.); 2 Key Laboratory of Forest Food Resources Utilization of Heilongjiang Province, Harbin 150040, China; 3 College of Life Sciences, Northeast Forestry University, Harbin 150040, China

**Keywords:** flavonoids, *N^ε^*-(carboxyethyl)lysine (CEL), trapping methylglyoxal, *C*-ring structures

## Abstract

This study investigated the effects of taxifolin (Tax), quercetin (Que), (+)-catechin (Cat) and luteolin (Lute) on the advanced Maillard reaction stage in the methylglyoxal-lysine (MGO-Lys) system. Since the four flavonoids share identical *A*- and *B*-ring structures, the inhibitory effects and molecular mechanisms of flavonoids with different *C*-ring structures on *N^ε^*-(carboxyethyl)lysine (CEL) formation were revealed. The results demonstrated that Cat exhibited the best inhibitory effect on CEL with an inhibition rate of 53.78%, while Lute showed the lowest inhibition rate of 3.97%. The flavonoids (i.e., Tax, Que, Cat and Lute) inhibited the formation of non-fluorescent CEL, where hydroxylation at C^3^ on the *C*-ring favored the enhancement of the inhibitory effect of the flavonoids on CEL, while the C^2^-C^3^ double bond and the carbonyl group at the C^4^ position reduced their inhibitory ability. The alkaline environment favored the enhancement of the inhibition of CEL by Tax, Que, Cat and Lute. Notably, Tax, Que, Cat and Lute can inhibit CEL formation by competitively capturing MGO to form mono- or di-adducts and reducing lysine consumption. This study provides innovative strategies and a theoretical foundation for developing effective CEL inhibitors in food thermal processing.

## 1. Introduction

Advanced glycation end products (AGEs) are complex compounds generated through Maillard reactions between carbonyl and amino groups during food processing. Diet-derived *N^ε^*-carboxyethyllysine (CEL), one of the representative AGEs, is prevalent in thermally processed foods and accumulated in humans through dietary intake [1,2]. Current evidence demonstrates that CEL activates RAGE-mediated oxidative stress and inflammatory pathways and has been closely associated with chronic pathologies, including diabetic nephropathy, atherosclerosis, and neurodegenerative diseases [3,4]. However, CEL generation is difficult to entirely prevent in current food processing, and conventional inhibition strategies (i.e., chemical additives or process optimization) are limited by safety issues or cost-effectiveness. Therefore, it is necessary to develop efficient and safe natural inhibitors to inhibit CEL formation or its metabolic pathways.

As naturally occurring bioactive compounds in plants, flavonoids have drawn more attention due to their biological activities, such as their anti-diabetic, antioxidant, anti-inflammatory and anticancer activities [5,6,7,8]. Furthermore, flavonoids have been shown to inhibit or mitigate the formation of Maillard reaction products in food model systems [9,10]. Current research on CEL primarily focuses on flavonoid-mediated MGO capture to inhibit CEL generation. Li et al. revealed that the phenolic hydroxyl group at C^3^ of the *C*-ring hindered flavonoid-methylglyoxal (MGO) adduct formation, thereby reducing CEL inhibition [11]. Chen et al. reported that the C^2^-C^3^ double bond significantly enhanced MGO capture capacity and CEL inhibition, while the C^4^ carbonyl group markedly impeded flavonoid binding with Amadori rearrangement products [12]. However, other studies have indicated that flavonoids with the C^3^ hydroxyl group exhibited stronger MGO capture capacity, whereas the C^2^-C^3^ double bond reduced the inhibitory effect of flavonoids on the end products of the Maillard reaction [13,14]. These conflicting findings highlight the need for further mechanistic studies to clarify the precise structure–activity relationship of *C*-ring modifications in flavonoid-mediated CEL inhibition.

**Figure 1:**
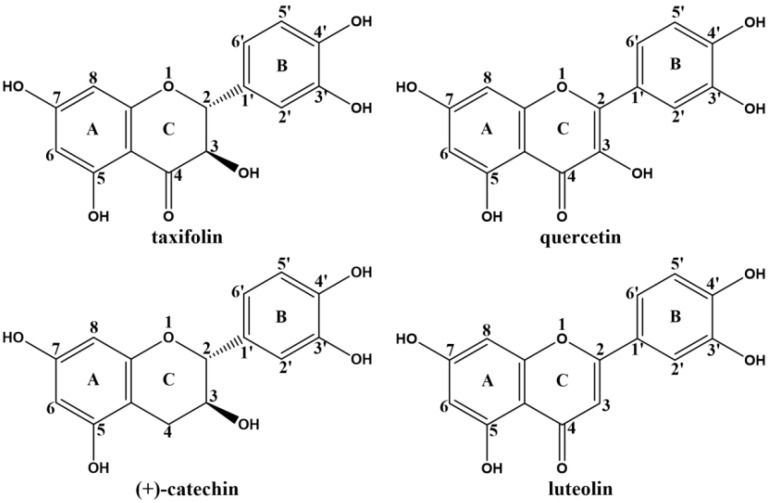
Structure of Tax, Que, Cat and Lute. The numbers represent the atomic sites of the flavonoids.

Based on the above research questions, this study systematically investigated four structurally distinct flavonoids, i.e., taxifolin (Tax), quercetin (Que), (+)-catechin (Cat) and luteolin (Lute) (Figure 1), on the inhibition of the formation of CEL in methylglyoxal-lysine (MGO-Lys) model system. Firstly, the changes in CEL content in the system and the effect of pH on the inhibition of CEL by Tax, Que, Cat and Lute were quantitatively analyzed by high-performance liquid chromatography with fluorescence detection (HPLC-FLD). Secondly, the competition mechanism of four flavonoids on CEL formation was investigated based on the changes in MGO and lysine content in the system. Finally, ultra performance liquid chromatography (UPLC) and mass spectrometry (MS) were employed to elucidate the direct trapping ability of the four flavonoids for MGO. This study aims to theoretically reveal the relationship between the molecular structure of flavonoids and their mechanism of inhibiting CEL formation.

## 2. Results and Discussion

### 2.1. Effects of Flavonoids on the Formation of CEL

**Figure 2:**
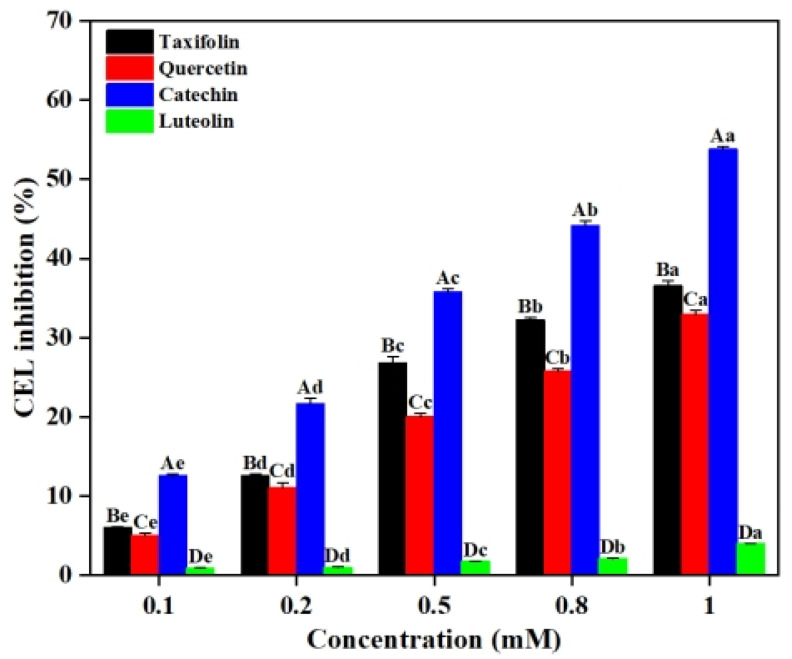
Inhibition rate of CEL in Lys-MGO simulation system by different concentrations of Tax, Que, Cat and Lute at pH 7.4. Different letters on the histograms represent significant differences (*p* < 0.05).

In the Lys-MGO system simulating the late stage of the Maillard reaction, CEL formation follows a classical carbonyl-amine cross-linking mechanism. The ε-amino group of lysine functions as a nucleophile to attack the carbonyl carbon of MGO, yielding an unstable intermediate Schiff base. This intermediate subsequently undergoes Amadori rearrangement to produce stable CEL [15]. As shown in Figure 2, the flavonoids (i.e., Tax, Que, Cat and Lute) all exhibited significant dose-dependent inhibition of CEL formations. When the concentrations of Tax, Que, Cat and Lute increased to 1 mM, the inhibition rates of CEL reached 36.55%, 32.91%, 53.78% and 3.97%, respectively. This may be due to the increased probability of binding of flavonoids to the Maillard reaction intermediates at higher concentrations [16]. Notably, the inhibitory effects on CEL formations from the flavonoids were Cat > Tax > Que > Lute, regardless of the flavonoid concentrations, as observed in Figure 2. Considering the four flavonoids have the same *A*- and *B*-rings, it can be speculated that hydroxylation at C^3^ on the *C*-ring enhanced the inhibitory effect of flavonoids on CEL, while the C^2^-C^3^ double bond and the carbonyl group at C^4^ reduced their inhibitory ability.

### 2.2. Effects of pH on CEL Formation

**Figure 3:**
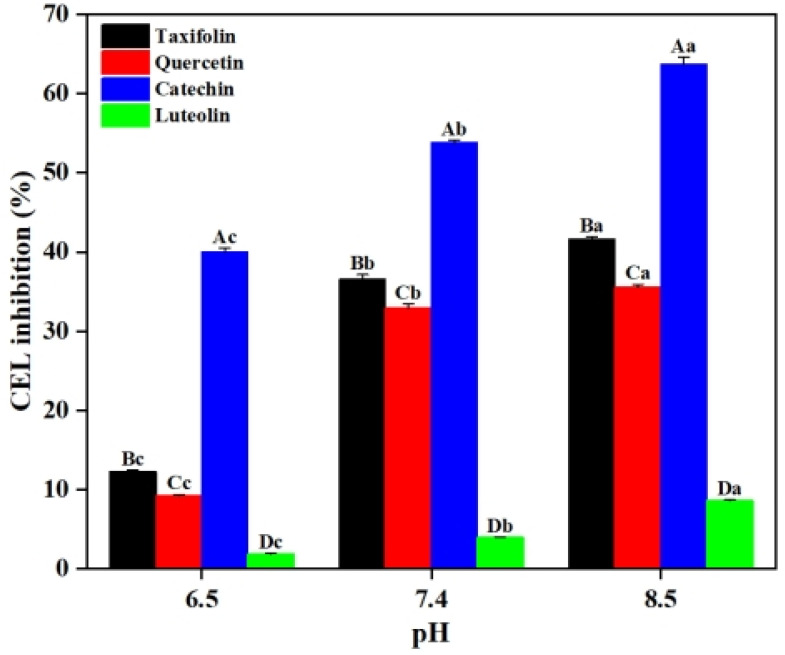
Effect of pH on the inhibition of CEL formation by Tax, Que, Cat and Lute. Capital letters represent significant differences between different samples at the same pH, while lowercase letters represent significant differences between different pH values of the same sample.

In order to show that the pH of the system has a modulating effect on the inhibition of CEL by flavonoids, the inhibition of CEL by the four flavonoids (i.e., Tax, Que, Cat and Lute) at different pH was determined in this section. As shown in Figure 3, when the pH of the system was 6.5, 1 mM of Tax, Que, Cat and Lute exhibited CEL inhibition rates of 12.26%, 9.27%, 35.02% and 1.90%, respectively. With pH increased to 8.5, the inhibition rates of CEL by Tax, Que, Cat and Lute reached 41.59%, 35.53%, 63.74% and 8.64%, respectively (*p* < 0.05). These results indicated that higher pH was conducive to the inhibitory effect of flavonoids on CEL. This phenomenon may be attributed to how the alkaline environment enhanced the deprotonation of flavonoids and increased their nucleophilicity, thereby improving the ability to capture MGO [17,18]. Therefore, the ability of Tax, Que, Cat and Lute to inhibit CEL formation was enhanced by increasing the pH of the system.

### 2.3. Effect of Flavonoids on MGO

**Figure 4:**
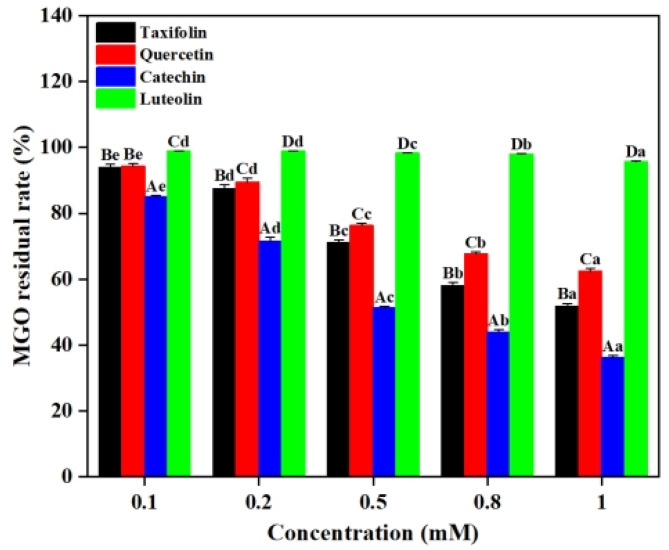
Effect of different concentrations of Tax, Que, Cat and Lute on the MGO content in the Lys-MGO system at pH 7.4. Capital letters represent significant differences between different samples at the same pH, while lowercase letters represent significant differences between different pH values of the same sample.

In the Lys-MGO-flavonoid system, the decrease in MGO content can be attributed to two competitive pathways. On one hand, MGO reacts with flavonoids via electrophilic substitution to form stable covalent adducts [19]. On the other hand, MGO directly interacts with lysine to generate CEL [20]. Consequently, quantifications of the remaining MGO in the systems after reactions reveal the efficacy of flavonoids in suppressing the formation of CEL. As shown in Figure 4, 0.1 mM of Tax, Que, Cat and Lute resulted in MGO residual rates of 93.97%, 94.33%, 85.08% and 98.99%, respectively. When the concentrations of flavonoids were 0.8 mM, Tax, Que, Cat and Lute demonstrated MGO residual rates of 58.09%, 67.68%, 44.00% and 98.09%, respectively. These results demonstrated that increasing concentrations of Tax, Que, Cat and Lute significantly enhanced their MGO capture capacities. Such observations agreed with previous studies reported. Jiao et al. found that a positive correlation between catechin concentration and its inhibition efficiency against glyoxal (GO), leading to effective reduction of *N^ε^*-(carboxymethyl)lysine (CML) levels [21]. It was found that Cat exhibited the strongest MGO capture capacity, followed by Tax. The observed difference may be explained by the strong electronegativity of the carbonyl oxygen atom on C^4^ leading to the migration of π-electrons on the *A*-ring to the *C*-ring, weakening the electron cloud density of C^6^ and C^8^, consequently decreasing their nucleophilicity and resulting in weaker reactivity of Tax with Maillard reaction intermediates compared to Cat [12]. Comparative analyses revealed that the magnitude of the capture ability of the four flavonoids on MGO was Cat > Tax > Que > Lute. These results confirmed the existence of competitive reaction mechanisms in the Lys-MGO-flavonoid system, where flavonoids inhibit CEL formation by competitively capturing MGO. Such significant capture ability might account for the observations that CEL inhibition rates increased in the reaction system with flavonoids, as shown in Figure 2.

### 2.4. Effect of Flavonoids on Lysine

**Figure 5:**
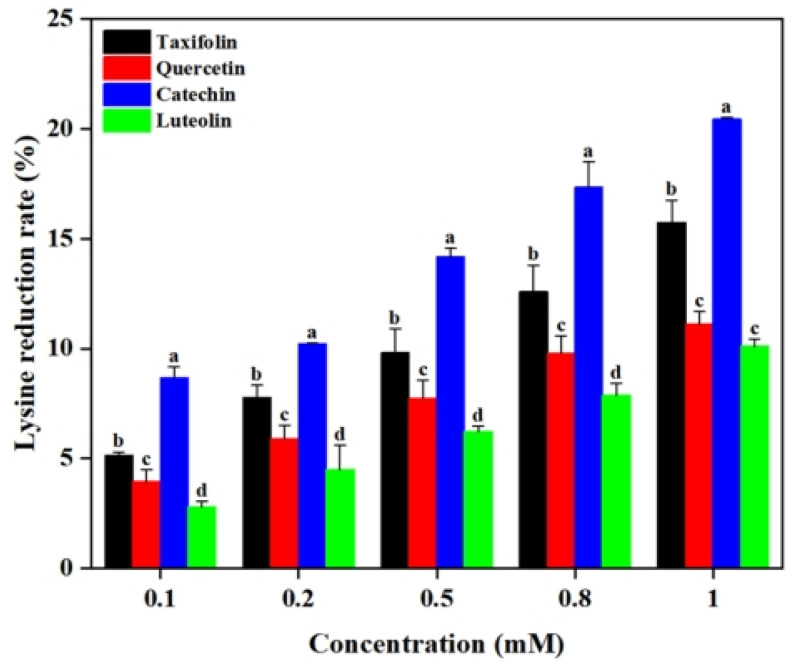
Effect of Tax, Que, Cat and Lute on Lysine content in the Lys-MGO reaction system at pH 7.4. (a, b, c and d indicate statistical significance).

Lysine serves as a key precursor substance for generating CEL in the late stage of the Maillard reaction, and its consumption level is an important indicator for evaluating the reaction progress [22]. The concentration of lysine after MGO reacting with lysine decreased from 2 mM to 1.42 mM (not presented in the figure) without the addition of flavonoids, indicating that alkaline environments favored the reaction between reductive amino groups and aldehyde groups to form Schiff bases, and subsequently leading to CEL generation [23]. However, as depicted in Figure 5, Tax, Que, Cat and Lute all significantly reduced the content of lysine reacting with MGO in the system; 0.1 mM of Tax, Que, Cat and Lute resulted in lysine reduction rates of 5.13%, 3.94%, 8.67% and 2.79%, respectively. When the concentration of Tax, Que, Cat and Lute reached 1 mM, the lysine reduction rates was increased to 15.74%, 11.12%, 20.44% and 10.11%, respectively. Such observations suggested that the addition of flavonoids to the Lys-MGO system enhanced their MGO capture ability to prevent lysine from attacking by MGO, thereby inhibiting the reaction between lysine and MGO to generate CEL, which was consistent with their inhibitory effects on CEL formation, as shown in Figure 2 [24].

### 2.5. Interactions Between Flavonoids and MGO

#### 2.5.1. Capture Capacity of Flavonoids on MGO

**Figure 6:**
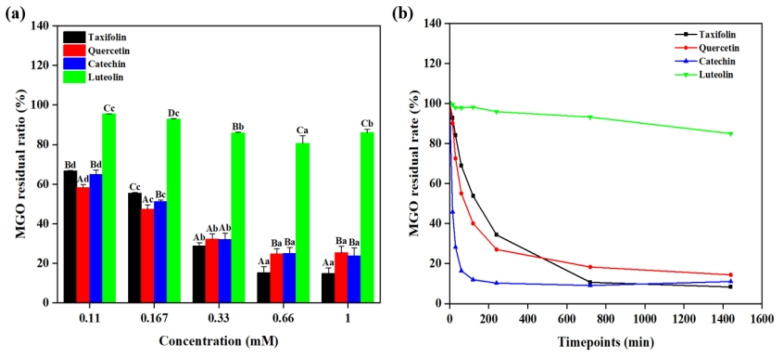
Capture effect of MGO by Tax, Que, Cat and Lute at different concentrations at 37 °C (**a**); capture effect of MGO by Tax, Que, Cat and Lute at different times, respectively (**b**). Capital letters indicate significant differences between different samples at the same pH, while lowercase letters indicate significant differences between different pH values of the same sample.

To further confirm whether the decreased MGO levels resulted from the direct capture effect of flavonoids on MGO, we investigated the effects of four flavonoids (Tax, Que, Cat and Lute) at different concentrations and reaction times on MGO residual rates. From Figure 6a, as the flavonoid concentrations increased from 0.11 mM to 0.66 mM, Tax, Que, Cat and Lute progressively reduced the MGO residual rate. At flavonoid concentrations of 0.11 mM and 0.167 mM, Que demonstrated higher MGO capture capacity than Tax, Cat and Lute. A possible explanation for this result was that the capture ability of MGO by flavonoids varies depending on concentration ratios [25]. On the other hand, the unsaturated C^2^-C^3^ double bond in Que enhanced the near-planarity of the *B*- and *C*-rings, which facilitated faster interaction between Que and MGO molecules [26].

As shown in Figure 6b, the MGO residual rates in the system of Tax, Que, Cat and Lute gradually decreased with the prolonged of reaction time. After 24 h of reaction, the residual rates of MGO in the reaction systems of Tax, Que, Cat and Lute were 8.33%, 14.39%, 11.04% and 84.99%, respectively. Throughout the reaction process, the MGO capture capacity of these flavonoids followed the order Tax > Cat > Que > Lute. Lute consistently demonstrated lower MGO capture capacity than Que and Tax, indicating that the absence of both C^2^-C^3^ double bond and C^3^ hydroxyl group on the *C*-ring reduced its MGO capture capacity [27]. Compared with Que, Tax showed greater MGO capture capacity, further confirming that the C^2^-C^3^ double bond on the *C*-ring decreased MGO capture capacity. Comparative analysis between Tax and Cat revealed that the C^4^ carbonyl group on the *C*-ring reduced MGO capture capacity. Zhu et al. investigated the effect of the *C*-ring structure of flavonoids on the capture efficiency of MGO under the same reaction conditions (pH = 7.4, 37 °C) [28]. Their results demonstrated that the double bond at the C^2^-C^3^ positions of the *C*-ring significantly reduced the capture efficiency of MGO by flavonoids, while the introduction of the hydroxyl group at the C^3^ position effectively enhanced their capture efficiency. These findings showed complete consistency with the *C*-ring structural regulation patterns revealed in this study.

#### 2.5.2. Product Identification of Flavonoids and MGO

The high MGO capture capacity may be attributed to the formation of adducts between the flavonoids and MGO, some of which were identified in structure using UPLC-MS/MS.

##### Identification of the Reaction Products of Taxifolin and MGO

**Figure 7:**
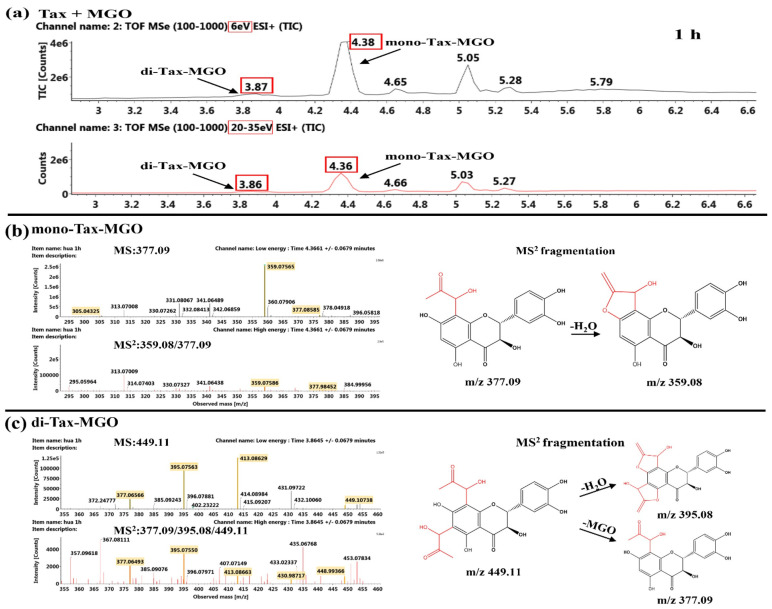
TIC chromatogram (**a**) and MS^n^ (*n* = 1–2) spectra and MS^2^ fragmentation of mono-Tax-MGO (**b**) and di-Tax-MGO (**c**) after 1 h of reaction of Tax and MGO.

The Tax adducts of mono- and di-Tax-MGO were obtained after 1 h of reaction of Tax and MGO, as shown in Figure 7. Two Tax-MGO adducts were initially identified with *m*/*z* 377.09 [M+H]^+^ and *m*/*z* 449.11 [M+H]^+^, which were 72 and 144 mass units higher than Tax (*m*/*z* 305.05 [M+H]^+^). Such identification of Tax-MGO adducts agreed with the observation of Zhang et al. [29]. The retention times of these two adducts corresponded to 4.38 min and 3.87 min in Figure 7a. After 24 h reaction of Tax and MGO, mono-Tax-MGO was still the main product, followed by di-Tax-MGO (Figure S1). Additionally, it can be seen from Figure 7b that the MS^2^ fragment at 359.08 [M–18+H]^+^ was characterized due to loss of an H_2_O of the mono-Tax-MGO adduct. In a similar manner, the MS^2^ fragment at 395.08 [M−36+H]^+^ was derived from loss of two H_2_O of the di-Tax-MGO adduct, as shown in Figure 7c.

##### Identification of the Reaction Products of Quercetin and MGO

**Figure 8:**
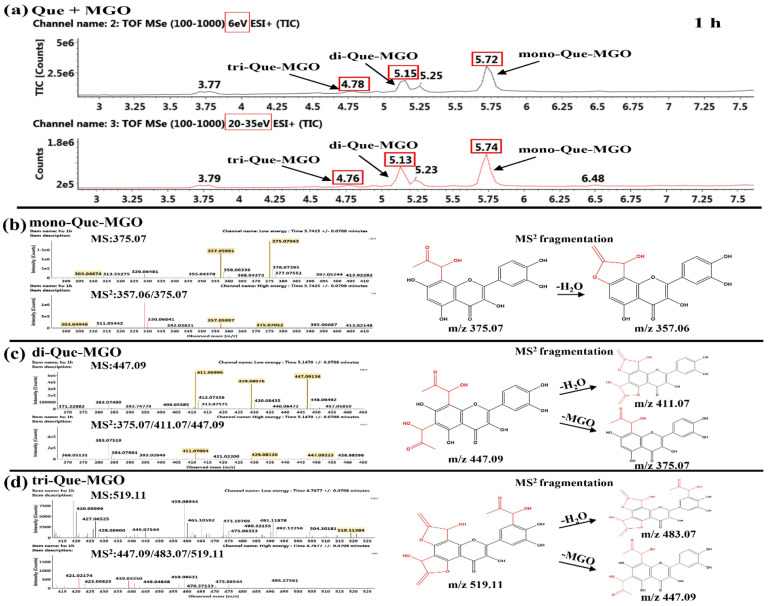
TIC chromatogram (**a**) and MS^n^ (*n* = 1–2) spectra and MS^2^ fragmentation of mono-Que-MGO (**b**), di-Que-MGO (**c**) and tri-Que-MGO (**d**) after 1 h of reaction of Que and MGO.

As shown in Figure 8, the existences of mono-, di-, and tri-Que-MGO adducts were tentatively characterized by typical MS^2^ fragments at *m*/*z* 375.07 [M+H]^+^, *m*/*z* 447.09 [M+H]^+^, and *m*/*z* 519.11 [M+H]^+^ after 1 h of reaction of Que and MGO. Our results were in agreement with the findings of Liu et al., who found that in addition to the *A*-ring, the *B*-ring of Que had the capacity to capture MGO to form the tri-adduct [30]. The MS^2^ fragment at 357.06 [M−18+H]^+^ was found due to the loss of a H_2_O of mono-Que-MGO adduct (Figure 8b), and the MS^2^ fragment at 411.07 [M−36+H]^+^ corresponded to the loss of two H_2_O in di-Que-MGO adduct (Figure 8c). Similarly, the MS^2^ fragments at *m*/*z* 501.09 [M−18+H]^+^ and *m*/*z* 483.07 [M−36+H]^+^ indicated that the tri-Que-MGO at *m*/*z* 519.11 [M+H]^+^ had the same fragmentation pattern as shown in Figure 8d. The retention times of these three adducts were detected at 5.72 min, 5.15 min and 4.78 min in the system, as shown in Figure 8a. The elution profiles matched previously reported flavonoid separation characteristics, demonstrating increased molecular polarity leading to reduced retention times [31]. Additionally, these characteristic product peaks exhibited significant attenuation accompanied by emerging impurity peaks after 24 h reaction, as shown in Figure S2, potentially resulting from either Que-MGO adduct instability or intermolecular cyclization [32]. The observed instability and low production of tri-Que-MGO adduct implied that it was more difficult for the reaction to occur, thus reducing the ability to inhibit CEL.

##### Identification of the Reaction Products of Catechin and MGO

**Figure 9:**
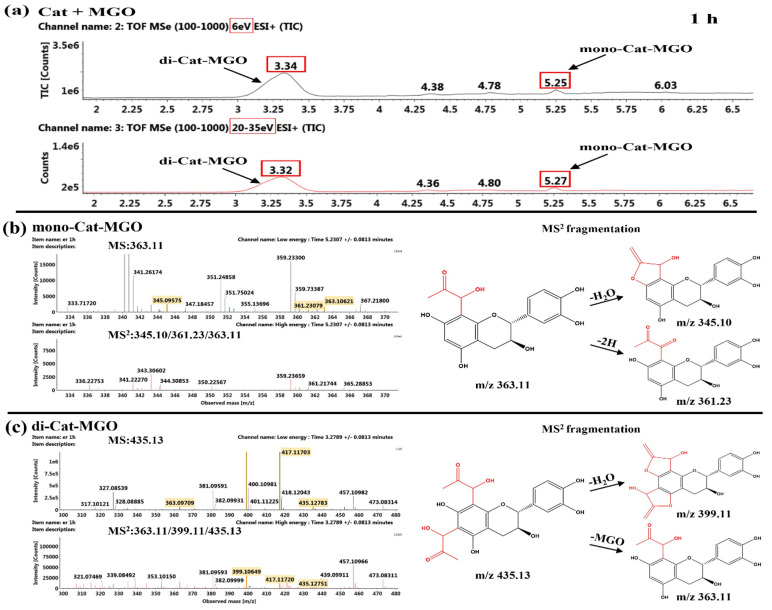
TIC chromatogram (**a**) and MS^n^ (*n* = 1–2) spectra and MS^2^ fragmentation of mono-Cat-MGO (**b**) and di-Cat-MGO (**c**) after 1 h of reaction of Cat and MGO.

As shown in Figure 9, the observed MS^2^ fragments at *m*/*z* 363.11 [M+H]^+^ and 435.13 [M+H]^+^ after 1 h of reaction of Cat and MGO, which were 72 and 144 mass units higher than that of Cat at *m*/*z* 291.08 [M+H]^+^, indicating they were mono-Cat-MGO adduct and di-Cat-MGO adduct. Such results agreed with the observation of Sang et al., who found that C^6^ or C^8^ of the *A*-ring was the major active site for capturing reactive dicarbonyl compounds [33]. Additionally, the retention times of these two adducts were observed at 5.25 min and 3.34 min as shown in Figure 9a, respectively. As depicted in Figure S3, these characteristic product peaks significantly decreased after 24 h reaction, likely due to oxidative degradation or intramolecular cyclization of the mono-Cat-MGO and di-Cat-MGO adducts in the system [33]. It was found that the MS^2^ fragment at *m*/*z* 345.10 [M−18+H]^+^ corresponded to the loss of a H_2_O of mono-Cat-MGO adduct (Figure 9b). Similarly, the MS^2^ fragment ions at *m*/*z* 417.12 [M−18+H]^+^ and *m*/*z* 399.11 [M−36+H]^+^ demonstrated that the di-Cat-MGO at *m*/*z* 435.13 [M+H]^+^ underwent an identical fragmentation pattern (Figure 9c). Furthermore, the observed MS^2^ fragment at *m*/*z* 361.23 [M−H]^+^) was two mass units lower than mono-MGO-Cat adduct, as shown in Figure 9b, suggesting this new product may be the mono-MGO-Cat-quinone.

##### Identification of the Reaction Products of Luteolin and MGO

**Figure 10:**
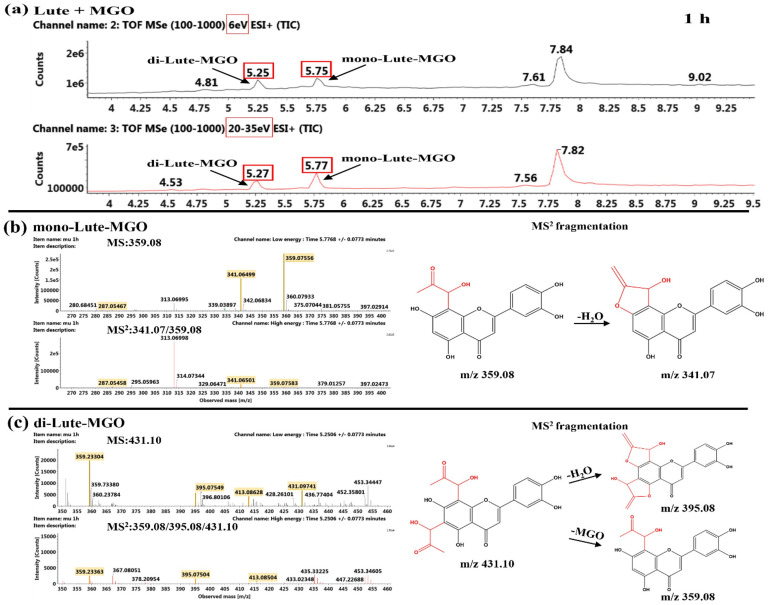
TIC chromatogram (**a**) and MS^n^ (*n* = 1–2) spectra and MS^2^ fragmentation of mono-Lute-MGO (**b**) and di-Lute-MGO (**c**) after 1 h of reaction of Lute and MGO.

As shown in Figure 10, the appearance of mono- and di-Lute-MGO adducts was tentative recognized by characteristic MS^2^ fragments at *m*/*z* 359.08 [M+H]^+^ and *m*/*z* 431.10 [M+H]^+^ after 1 h of reaction between Lute and MGO, which was consistent with that reported for Lute–MGO in the literature [34]. It was found that the MS^2^ fragment at *m*/*z* 287.05 [M−72+H]^+^ corresponded to the loss of a MGO of mono-Lute-MGO adduct (Figure 10b). The MS^2^ fragment at 341.07 [M−18+H]^+^ was identified due to the loss of a H_2_O of mono-Lute-MGO adduct (Figure 10b), and the MS^2^ fragments at *m*/*z* 413.09 [M−18+H]^+^ and *m*/*z* 395.08 [M−36+H]^+^ corresponded to the loss of one and two H_2_O of di-Lute-MGO (Figure 10c), respectively. Furthermore, these characteristic product peaks were detected at 5.75 min and 5.25 min, as shown in Figure 10a, respectively. After 24 h of reaction between Lute and MGO, mono-Lute-MGO and di-Lute-MGO were still the major products (Figure S4).

Previous studies reported that flavonoids primarily captured α-dicarbonyl compounds at the C^6^ or C^8^ position of the *A*-ring [35]. It can be seen that Tax, Que, Cat and Lute initially reacted with MGO at C^6^ or C^8^ to form mono-MGO adducts, followed by subsequently capturing another MGO molecule at the available *A*-ring site. Although the tri-Que-MGO adduct was detected in the Que-MGO system, it can be inferred from the combination of UPLC-MS/MS data and experimental results that the presence of the C^2^-C^3^ unsaturated bond in the *C*-ring of the flavonoids weakened the stability of the adducts formed with MGO, thus decreasing the inhibitory effect on CEL formation. These findings confirmed that flavonoids can inhibit CEL formation by competitively capturing MGO.

## 3. Materials and Methods

### 3.1. Materials and Chemicals

Four flavonoids (purity ≥96%, Taxifolin, quercetin, (+)-catechin and luteolin were labeled as Tax, Que, Cat and Lute, respectively) were purchased from Shanghai Yuanye Biotechnology Co., Ltd. (Shanghai, China). Methylglyoxal (MGO, 40% in water), L-lysine, o-phenylenediamine (OPD), 2-mercaptoethanol (βME) (98%, GC), o-phthalaldehyde (OPA), formic acid (LC-MS grade), methanol (LC-MS grade) and ethanol (LC-MS grade) were obtained from Ron Reagent Co., Ltd. (Shanghai, China). All other chemicals utilized in this research were of analytical-grade standards. All experimental procedures were conducted using ultra-pure water produced by a Milli-Q water purification system (Millipore, Billerica, MA, USA).

### 3.2. Preparation of MGO-Lys-Flavonoid Model

The MGO-Lys-flavonoid model system was prepared and slightly optimized with reference to Qin et al. [36]. Firstly, lysine (6 mM) and MGO (1 mM) stock solutions were prepared in phosphate-buffered saline (PBS, 20 mM, pH 7.4). Then, a MGO-Lys reaction system was established by mixing 6 mM of lysine and 1 mM of MGO in a 1:1 volume ratio. Subsequently, the stock solutions of flavonoids (i.e., 10 mM of Tax, Que, Cat and Lute in ethanol) were individually introduced into the system to attain a series of solutions with the flavonoid concentrations of 0.1, 0.2, 0.5, 0.8 and 1 mM, respectively. The reaction mixtures were heated at 80 °C for 5 min followed by rapid cooling in an ice-water bath to arrest further reaction progress. The samples were collected and stored at −20 °C for subsequent analysis. The blank control group was substituted with the same volume of ethanol solution instead of the flavonoid solution.

### 3.3. Determination of CEL

The variation in CEL in the system was determined according to the previously reported pre-column derivatization HPLC method with minor modifications [37]. The sample treatment procedure was as described below: 0.75 mL of reaction mixtures (as prepared in Section 3.2) and the blank control groups were separately mixed with 2 mL of sodium borate buffer solution (0.2 M pH 9.4) and 1 mL of sodium borohydride (dissolved in 0.1 M NaOH) in a centrifuge tube, and then allowed to reduce at room temperature for 4 h. Subsequently, 0.5 mL of reduced sample solutions were thoroughly combined with 2 mL OPA derivatization reagent, and immediately passed through a 0.45 µm filtration membrane for liquid phase analysis.

Preparation of OPA derivatization reagent: first, 10 mg of OPA was dissolved in 2 mL of methanol to prepare the OPA solution: 1 mL of OPA solution was then mixed with 8 μL of βME and 3.992 mL of borate buffer (0.2 M boric acid and 0.2 M NaOH, pH 9.9) to obtain the derivatization reagent [38].

The chromatographic conditions were as follows: the chromatographic column was a Venusil ASB C18 column (4.6 × 250 mm, 5 μm; Bona-Agela Technology Co., Tianjin, China), the mobile phase A was 0.1% aqueous trifluoroacetic acid, and the mobile phase B was pure chromatographic acetonitrile at a flow rate of 0.8 mL/min. The injection volume was set at 10 μL, with fluorescence detection at excitation (λ_ex_) and emission (λ_em_) wavelengths of 340 nm and 455 nm, respectively. The gradient elution procedure was as follows: 0–5 min, 5–70% B; 5–17 min, 70–70% B; 17–18 min, 70–5% B; 18–28 min, 5–5% B. The content of CEL in the samples was calculated from the standard curve of CEL (0.05–2 mM; y = 1,165,822.76623x + 4830.78377, R^2^ = 0.9992). The CEL inhibition rate was calculated by using the following formula:

(1) $$\mathrm{CEL}\;\mathrm{inhibition}\;\mathrm{rat}e=\frac{{\mathrm{CEL}}_{\mathrm{control}}-{\mathrm{CEL}}_{\mathrm{sample}}}{{\mathrm{CEL}}_{\mathrm{control}}}\text{×}100\%$$

In the formula, CEL_(control)_ was the content of CEL in the blank control groups and CEL_(sample)_ was the content of CEL in the flavonoid sample groups.

### 3.4. Effects of pH on the Formation of CEL

The Lys-MGO-flavonoid model systems were prepared in 20 mM PBS buffer at different pH (i.e., 6.5, 7.4 and 8.5) values with all four flavonoids at a final concentration of 1 mM [17]. All reaction systems were heated at 80 °C for 5 min. The blank control groups were prepared with ethanol solutions. The CEL derivatives were detected by HPLC following the established analytical conditions.

### 3.5. Determination of MGO

The content of MGO produced in the MGO-Lys system was determined according to a previously reported and modified UPLC method [39]; 1 mL of control groups and experimental groups were separately mixed with 0.25 mL of OPD (20 mM) and stirred with a vortexer for 5 s. All samples were heated at 37 °C for 1 h for derivatization. All samples were passed through a 0.22 µm filter membrane before analysis and then injected into an e2695 UPLC system (Waters, Milford, MA, USA). The system was equipped with an ACQUITY BEH C18 column (2.1 × 100 mm, 1.7 μm; Waters Technologies Co., Ltd., Milford, MA, USA). The conditions for the detection of MGO were as follows: the mobile phase consisted of ultrapure water containing 0.1% formic acid (mobile phase A) and pure chromatographic grade methanol (mobile phase B) at a flow rate of 0.21 mL/min. The gradient elution program was as follows: 0–2.08 min, 5% B; 2.08–3.28 min, 5–50% B; 3.28–8.48 min, 50–50% B; 8.48–8.88 min, 50–90% B; 8.88–12.08 min, 90–90% B; 12.08–13 min, 90–5% B; 13–14 min, 5–5% B. The injection volume was 5 µL, and the UV detection wavelength was 315 nm. The content of MGO in the samples was calculated from the standard curve of 2-methylquinoxaline (0.01–1 mM; y = 5,871,593.61926x − 2185.85774, R^2^ = 0.9999). The MGO residual rate was calculated using the following equation:

(2) $$\mathrm{MGO}\;\mathrm{residual}\;\mathrm{rate}=\frac{{\mathrm{MGO}}_{\mathrm{sample}}}{{\mathrm{MGO}}_{\mathrm{control}}}\text{×}100\%$$

In the formula, MGO_(control)_ is MGO content in the blank control groups and MGO_(sample)_ is MGO content in the flavonoid sample groups.

### 3.6. Determination of Lysine

Lysine was determined with reference to the previous method with slight modifications [40]; 0.2 mL of blank control and experimental group samples were mixed with 1 mL of OPA derivatization reagent and vortexed for 2 min, respectively. The solutions were then immediately filtered through 0.45 μm membranes into HPLC vials for analysis. The chromatographic conditions were as follows: the chromatographic column was a Venusil ASB C18 column (4.6 × 250 mm, 5 μm; Bona-Agela Technology Co., Tianjin, China). The mobile phase A was 0.1% aqueous trifluoroacetic acid, and the mobile phase B was pure chromatographic acetonitrile at a flow rate of 0.8 mL/min. The injection volume was 10 μL, with detection wavelengths of λ_ex_ = 340 nm and λ_em_ = 455 nm, and the gradient elution program was as follows: 0–5 min, 5–70% B; 5–17 min, 70–70% B; 17–18 min, 70–5% B; 18–28 min, 5–5% B. The lysine content in the samples was calculated according to the standard curve of lysine (0.05–2 mM; y = 1,121,611.92341x + 32,983.4061, R^2^ = 0.9989). The reduction rate of lysine in the system reacting with MGO after the addition of flavonoids was calculated by the following equation:

(3) $$\mathrm{Lys}\;\mathrm{reduction}\;\mathrm{rate}=\frac{{\mathrm{Lys}}_{\mathrm{control}}-{\mathrm{Lys}}_{\mathrm{sample}}}{{\mathrm{Lys}}_{\mathrm{control}}}\text{×}100\%$$

where Lys_(control)_ represented Lysine content in the blank control groups and Lys_(sample)_ represented Lysine content in the flavonoid sample groups.

### 3.7. Determination of MGO Capture Capacity by Flavonoids

MGO (0.33 mM) was combined with different concentrations of flavonoids in 20 mM PBS buffer (pH 7.4). The reaction solutions were incubated at 37 °C for 24 h. Subsequently, 1 mL of reaction mixtures were separately derivatized with 0.25 mL of OPD (20 mM) at 37 °C for 1 h. The resulting MGO-OPD adducts were quantified via UPLC using the previously described analytical method [41].

To study the ability of flavonoids to capture MGO at different reaction times, 0.5 mL of MGO (0.33 mM) was incubated with 0.5 mL of flavonoids (1 mM) in 20 mM PBS buffer (pH 7.4) for 0, 15, 30, 60, 120, 240, 720, 1440 min, respectively. At each time point, 8 μL of acetic acid was added to terminate the reaction in an ice bath. Subsequently, 0.25 mL of OPD (20 mM) was mixed and incubated for 1 h at 37 °C away from light to allow full derivatization [42]. The residual MGO concentration in the reaction mixtures was subsequently quantified using the established UPLC analytical protocol.

### 3.8. Identification of the Flavonoid-MGO Adducts

In total, 0.5 mL of MGO (3 mM) was mixed with 0.5 mL of flavonoids (1 mM) and incubated at 37 °C for 1 h and 24 h. Prior to analyses, the samples were subjected to filtration using a 0.22 μm membrane to eliminate particulate impurities and then injected into the injection vial. Flavonoid-MGO adducts were analyzed using ultra performance liquid chromatography–time-of-flight mass spectrometry (UPLC-Q-TOF-MS) (Waters Corporation, MA, USA) connected to an electrospray ionization (ESI) source [43].

The UPLC-MS conditions were as follows: the system was equipped with an ACQUITY BEH C18 column (2.1 × 100 mm, 1.7 μm; Waters Technologies Co., Ltd., USA). The injection volume was 1 µL, the column temperature was 45 °C, and the flow rate was 0.21 mL/min. The mobile phase A was ultrapure water containing 0.1% formic acid, and the mobile phase B was pure chromatographic acetonitrile. The gradient elution program was as follows: 0–6 min, 10–45% B; 6–14 min, 45–55% B; 14–15 min, 55–95% B; 15–17 min, 95–95% B; 17–18 min, 95–10% B; and 18–19 min, 10–10% B. The mass scan range was 100–1000 *m*/*z* in positive ion mode, with an ion source temperature of 135 °C, a desolvent gas temperature of 350 °C, a desolvent gas flow rate of 600 L/h, a capillary voltage of 3.00 kV, and a single scan time of 1 s.

### 3.9. Data Analysis

All determinations were repeated three times and the results were expressed as mean ± standard deviation. Data processing and graphical visualization were performed using Excel 2021 and Origin 2022. The data were analyzed for significance by one-way ANOVA using SPSS 26.0 software, followed by Duncan’s multiple range tests. The differences between the data were determined to be statistically significant at *p* < 0.05.

## 4. Conclusions

This study investigated the inhibitory effects of flavonoids with different *C*-ring structures on CEL formation and their underlying mechanisms. It was found that Tax, Que, Cat and Lute could significantly inhibit the formation of CEL in the Lys-MGO simulation system, and their inhibitory activities were in the following order: Cat > Tax > Que > Lute. Structure–activity relationship analysis of these four flavonoids revealed that the presence of the hydroxyl group at the C^3^ position markedly enhanced the inhibitory effect of the flavonoids on CEL, while the C^2^-C^3^ double bond and the carbonyl group at the C^4^ position reduced their inhibitory effect on CEL. Furthermore, the inhibitory effects of Tax, Que, Cat and Lute on CEL were significantly enhanced with the increase in pH of the system. UPLC-Q-TOF-MS analysis revealed that Tax, Que, Cat and Lute directly captured MGO to form mono- and di-adducts, while only Que produced detectable tri-adducts. It is worth noting that Tax, Que, Cat and Lute mainly inhibited CEL formation by competitively capturing MGO and attenuating the further attack of MGO on lysine in the system. Given the current scarcity of research on specific end-product CEL in foods and existing controversies regarding *C*-ring structure effects on flavonoid-mediated CEL inhibition, the current work, including the changes in CEL, the competitive inhibition among flavonoid–MGO–lysine, and the identification of products of flavonoids, and MGO might provide novel insights into the mechanism linking flavonoid structural variations to CEL inhibition. In particular, the established Lys-MGO model system provides novel findings for *C*-ring structural determinants in flavonoid-mediated CEL suppression pathways.

## Data Availability

The original contributions presented in this study are included in the article/Supplementary Material. Further inquiries can be directed to the corresponding author(s).

## Supplementary Materials

The following supporting information can be downloaded at https://www.mdpi.com/article/10.3390/ijms26125914/s1.
