# Supplementary material for: Effects of C-Ring Structural Differences on the Inhibition of Nε-(Carboxyethyl)lysine in the Methylglyoxal-Lysine System by Flavonoids
Source: Int J Mol Sci. 2025 Jun 19;26(12):5914. doi: 10.3390/ijms26125914 (PMC12193151; doi:10.3390/ijms26125914)
Supplement: Supplementary file 1 [file ijms-26-05914-s001.zip › ijms-3656500-supplementary.pdf]

Supplementary document

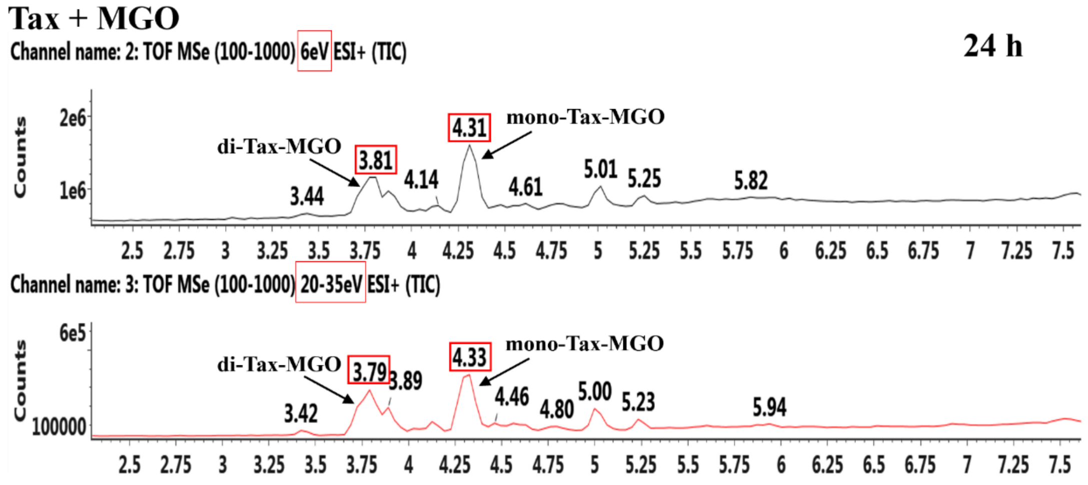

Figure S1. TIC chromatogram after reaction of Tax and MGO for 24 h at pH 7.4 and 37 °C.

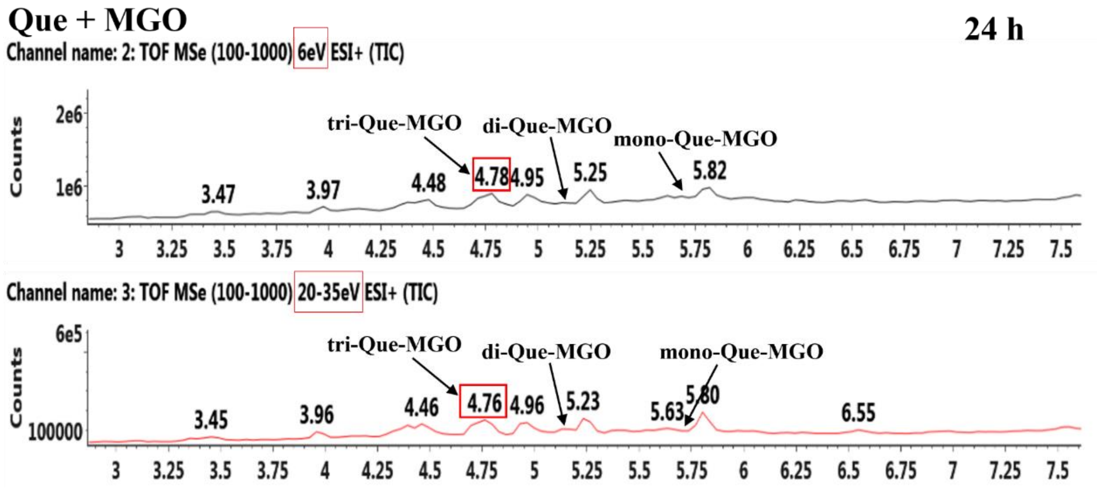

Figure S2. TIC chromatogram after reaction of Que and MGO for 24 h at pH 7.4 and 37 °C.

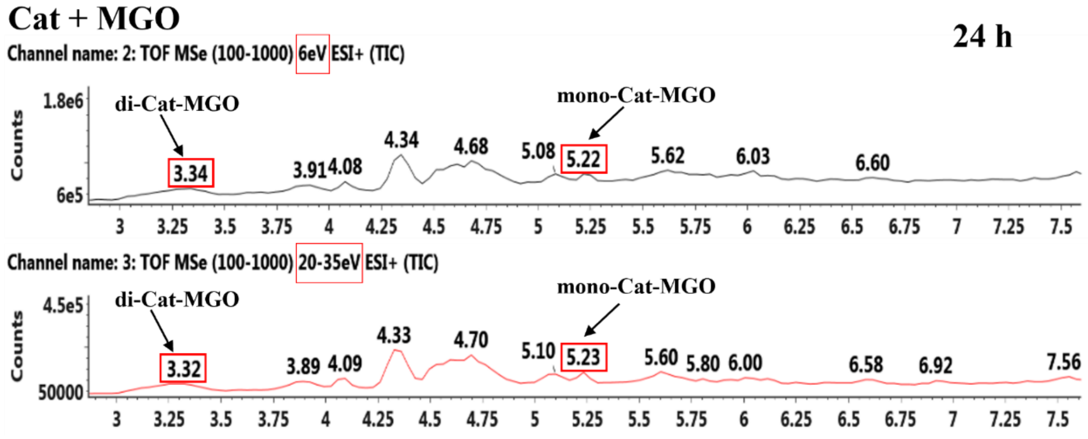

Figure S3. TIC chromatogram after reaction of Cat and MGO for 24 h at pH 7.4 and 37 °C.

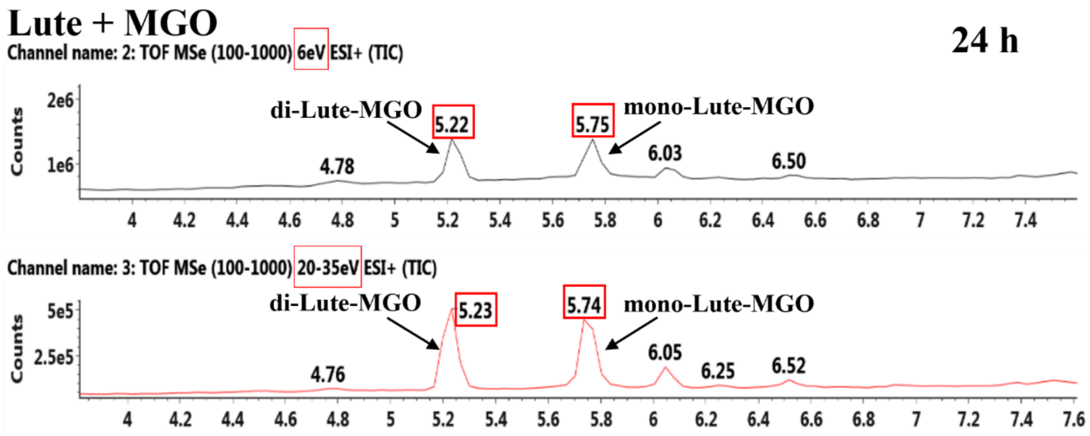

Figure S4. TIC chromatogram after reaction of Lute and MGO for 24 h at pH 7.4 and 37 °C.
